# A New Hypersonic Wind Tunnel Force Measurement System to Reduce Additional Bending Moment and Avoid Time-Varying Stiffness

**DOI:** 10.3390/s22072572

**Published:** 2022-03-27

**Authors:** Shichao Li, Zihao Liu, Fan Zhao, Hongli Gao

**Affiliations:** 1 China Aerodynamics Research and Development Center, Mianyang 621000, China; docterlsc9077@swjtu.edu.cn; 2 School of Mechanical Engineering, Southwest Jiaotong University, Chengdu 610031, China; zihaoliu0915@163.com (Z.L.); hongli_gao@swjtu.edu.cn (H.G.)

**Keywords:** wind tunnel force test, force measuring system, joint, dynamic modelling

## Abstract

In order to improve traditional hypersonic wind tunnel airframe/propulsion integrated aerodynamic testing technology for hypersonic vehicles, a new force measurement system called the aerodynamic force measuring support (AFMS) was designed. The AFMS effectively overcomes the defect that the traditional internal box-balance occupies a large amount of internal space in the aircraft test model, which makes the airframe/propulsion integrated aerodynamic test more difficult. The AFMS also alleviates the interference of the additional bending moment caused by the non-coincidence between the calibration center of traditional external box-balance and the gravity center of the aircraft test model, innovatively designing a convex structure in the joint part of the force measuring system. Furthermore, the AFMS effectively overcomes the time-varying stiffness of joints caused by test model vibration in hypersonic wind tunnel testing, which eventually leads to test errors. Compared with the traditional box-balance, the AFMS proposed in this study has sufficient design space. This ensures more thorough aerodynamic decomposition of the AFMS and less interference between channels, whilst also having the advantages of the large support stiffness of traditional box-balance. Thus, the AFMS provides a new technical path for airframe/propulsion integrated aerodynamic testing of air-breathing hypersonic vehicles in a hypersonic wind tunnel.

## 1. Introduction

An air-breathing hypersonic vehicle has a flight speed of up to 5 Ma, so it has good penetration and is difficult to intercept by an air defense system [1,2,3]. In addition, it adopts a scramjet engine as its propulsion system and has the advantages of high flight efficiency. This has made air-breathing hypersonic aircrafts a research hotspot in the current aviation field.

In order to design a vehicle with good aerodynamic performance and ensure its flight safety, it is necessary to reveal its aerodynamic characteristics accurately in the design stage, so as to guide the designers to optimize and modify it. Wind tunnel testing, Computational Fluid Dynamics (CFD), and aircraft test flights can achieve the above objective. At present, the aerothermal mechanism of hypersonic vehicles in high Mach number flight states is not clear, so it is difficult for CFD to accurately simulate the flow field state of air-breathing hypersonic vehicles during flight. The aircraft flight test technology is expensive, time-consuming, and has little effective data, so it is mainly used in the final validation stage of vehicle. However, wind tunnel testing has the advantages of low testing costs, repeated tests, and abundant tests, which are the key support technology for the research and development of air-breathing hypersonic vehicles [4,5,6,7].

The coupling between the propulsion system and the airframe in the air-breathing hypersonic vehicle is very strong. In order to accurately evaluate aerodynamic characteristics of hypersonic vehicles, it is imperative to carry out high-precision airframe/propulsion integrated aerodynamic wind tunnel testing. That is to say, in the process of wind tunnel testing, the scramjet needs ignition, and this is a frontier topic within the field.

There are many shortcomings in the application of traditional wind tunnel aerodynamic testing technology in hypersonic wind tunnels. (1) The bar-shaped balance adopts a cantilever support mode to the aircraft test model, which means its support stiffness is weak and it is therefore difficult to apply to the hypersonic wind tunnel for large-size model aerodynamic force tests [8]. (2) The box-balance can be divided into internal box-balance [9] and external box-balance [10,11,12,13]. The internal box-balance is mounted in the abdomen of the test model. It takes up a lot of space in the aircraft test model, which makes it impossible to install the propulsion system and difficult to carry out the aircraft/propulsion integrated aerodynamic test. The traditional external box-balance can solve the above deficiencies of the internal box-balance, but the center of gravity aircraft test model and the calibration center of the external box-balance do not overlap. In this case, additional bending moments will be generated, and interference between test channels is massive, which affects the test accuracy. Furthermore, the traditional external box-balance also has the disadvantages of large and complex equipment, high cost, and a long design and manufacturing cycle. (3) The suspension force measuring system places the aerodynamic testing device outside the test model [7,14,15]. Although it does not occupy the internal space of the test model, its installation is complicated and the support structure has great interference with the flow field, affecting the simulation accuracy of the real flow field. (4) The balance-support integrated device is a new force measurement technology that has just been proposed [16]. It integrates aerodynamic test components into the support of the aircraft model, which not only plays a supporting role but also realizes the decomposition and testing of the aerodynamic force received by the test model. However, the gravity center of the aircraft test model and the calibration center of the external box-balance do not overlap. In this case, additional bending moments will be generated, and interference between test channels is massive, resulting in impaired test accuracy. (5) The magnetic suspension balance uses magnetic force to fix the test model in the wind tunnel, so the flow field will not be disturbed. In addition, the aerodynamic test device is placed outside the test model and does not occupy the internal space of the test model [17,18]. However, this technology is not mature yet, and the support force is small, so it is difficult to apply to the hypersonic wind tunnel for large-size model aerodynamic force tests. Therefore, it is difficult for all the above aerodynamic testing techniques to carry out airframe/propulsion integrated aerodynamic tests of air-breathing hypersonic vehicle in a hypersonic wind tunnel.

To overcome these difficulties, this paper proposes a new aerodynamic force measurement technology that integrates the box-balance and the aircraft model support into a whole. This technology namely integrates aerodynamic force measurement functions into the aircraft test model support, and is called the aerodynamic force measuring support (AFMS). The AFMS has the following advantages: (1) The AFMS does not occupy the installation space of the propulsion system in the vehicle test model. Therefore, aircraft/propulsion integrated aerodynamic testing can be carried out without making any changes to the aircraft test model, which greatly reduces the test difficulty. (2) The calibration center of the AFMS coincides with the gravity center of the aircraft test model, which avoids the additional bending moment caused by the axial aerodynamic force acting on the aircraft test model. Therefore, the interference between test channels is effectively reduced and the testing accuracy is improved. (3) A series of cylindrical convex structures are designed on the joint between the aircraft test model and the AFMS, which can effectively overcome the time-varying stiffness characteristics of the AFMS caused by test model vibration during hypersonic wind tunnel testing, and effectively improves the testing accuracy of the AFMS in harsh vibration environments.

## 2. Testing Principle

**Figure 1:**
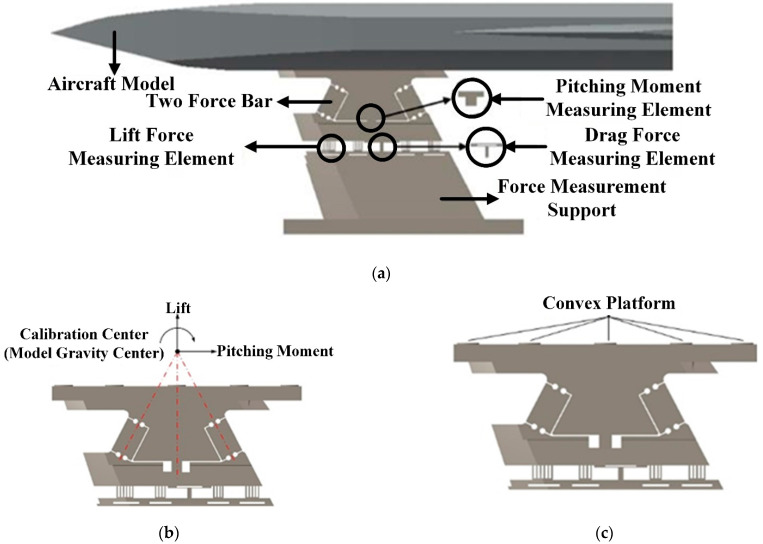
Three-dimensional model diagram of force measuring support. (**a**) The 3D model of AFMS. (**b**) The force measuring elements in AFMS. (**c**) The convex platforms in the joint of AFMS.

The AFMS proposed in this paper is a three-component aerodynamic testing system, which can simultaneously measure the axial force (thrust/drag force), lift force, and pitching moment acting on the aircraft test model. The 3D model, as shown in Figure 1a, is composed of thrust/drag force measuring elements, lift force measuring elements and pitching moment measuring elements, as shown in Figure 1b, which are, respectively, used to measure the axial force, lift force, and pitching moment received by the aircraft test model. Strain gauges are pasted on each element, and a Wheatstone bridge is formed. This converts the deformation of the force measuring element into resistance transformation of strain gauges, and, finally, into voltage output. Its testing principle is briefly as follows: in the process of hypersonic wind tunnel aerodynamic testing, the aircraft test model is affected by the thrust generated by the propulsion system and aerodynamic force. Thrust and aerodynamic force are transferred to the pitching moment measuring elements through two-force-bar and make it deformed, which causes the resistance strain gauge attached to the pitching moment measuring elements to change. A Wheatstone bridge outputs the resistance transformation in the form of voltage, so as to measure the pitching moment received by the aircraft test model. The lift and axial forces (the combined force of thrust and drag) applied to the aircraft test model are transferred downward to thrust/drag force measuring elements and lift force measuring elements, respectively, and the testing principle is the same as the pitching moment testing principle.

**Figure 2:**
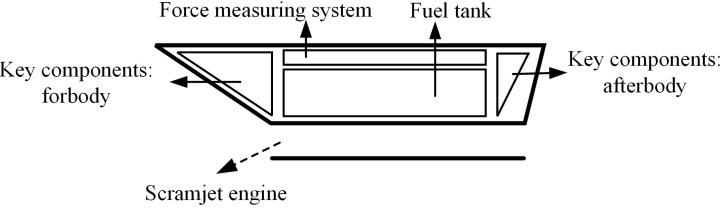
Inner space of air-breathing hypersonic vehicle.

The internal space layout of the air-breathing hypersonic vehicle test model is shown in Figure 2. Various key functional components, such as the propulsion system, fuel tank, fuel tubing, and electronics, occupy a large amount of space inside the aircraft test model, leaving limited installation space for the force-measuring system. Both the traditional bar-shaped balance and internal box-balance need to be installed inside the aircraft test model, usually occupying a large amount of internal space. This leads to space conflicts between balance and other functional components in the test model. The usual practice is to redesign the internal space of the test model and rearrange the functional parts, but this practice increases the difficulty and prolongs the test period, and is sometimes even ineffective. In order to solve this problem, a new aerodynamic force/engine thrust integrated testing technology, the aerodynamic force measuring support (AMFS), is proposed in this study, as shown in Figure 1a. It integrates the box-balance and the model support into a support frame with aerodynamic test function, meaning the aerodynamic test unit is integrated into the support frame. The design scheme has the following advantages:

(1) Aerodynamic testing equipment no longer occupies the internal space of the air-breathing hypersonic vehicle test model, which greatly reduces the difficulty of carrying out the airframe/propulsion integrated test of the air-breathing hypersonic vehicle test model;
(2) Because the AFMS is installed outside the test model, it does not occupy the test model’s internal space. Therefore, the AFMS is better than the traditional bar-shaped balance and internal box-balance technology. In this way, the force measuring elements can be designed and arranged more reasonably, and the aerodynamic force of the test model can be decomposed more thoroughly. Moreover, the interference among channels can be reduced, and the test accuracy can be improved;
(3) The support mode of the AFMS is the same as that of box-balance, which adopts either abdominal support or back support, so it has good support stiffness and strength. Therefore, it is suitable for carrying out aerodynamic test in a hypersonic wind tunnel;

## 3. Suppress the Interference of Axial Force Test Channel to Pitching Moment Test Channel

**Figure 3:**
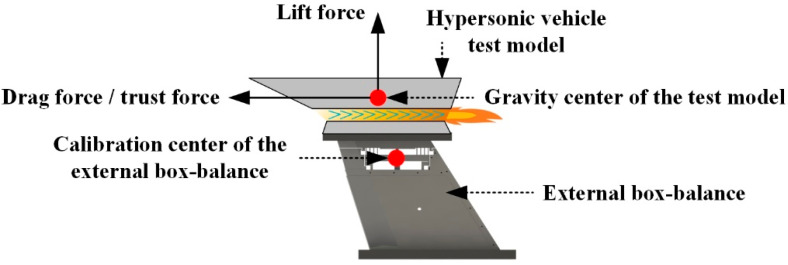
Three-dimensional model diagram of external box-balance.

**Figure 4:**
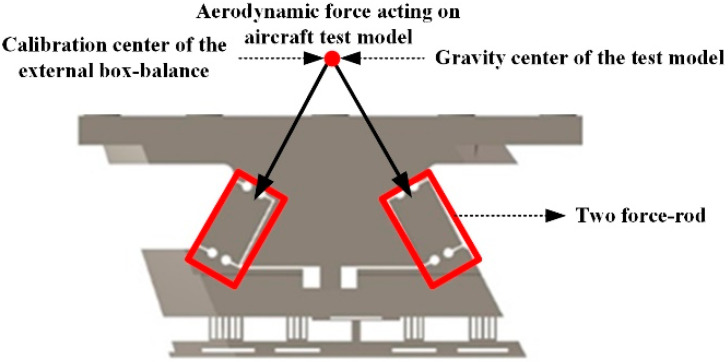
The force transmission path of aerodynamic force in the AFMS.

For the traditional external box-balance, the gravity center of the test model usually does not coincide with the calibration center of the balance, as shown in Figure 3. In this way, the axial force (drag force/thrust force) on the test model will generate an additional bending moment to the calibration center of the external box-balance, which will be measured together with the pitching moment on the model by the pitching moment test channel of the external box-balance. Thus, the interference error between channels increases. In order to improve the above defects, two-force rods were innovatively introduced into the AFMS in this study to avoid additional bending moment caused by axial force on the AFMS. The specific principle is as follows:

The axial force and lift force received by the aircraft test model are transmitted downward to the axial force test elements and lift force test elements through the pitching moment force measuring elements and two-force rods in the AFMS. Since two-force rods can only transmit axial force, as shown in Figure 4, the combined force of two two-force rods in the X direction is equal to the axial force received by the aircraft test model. Similarly, the combined force of two two-force rods and the pitching moment in the Y direction is equal to the lift force received by the aircraft test model. Since two-force rods can only transmit axial force, only the axial force bears the pitching moment of the aircraft test model. If the gravity center of the aircraft test model does not coincide with the calibration center of the AFMS, the axial force will generate additional bending moment. Since the AFMS has sufficient design space, the angle of the two tow-force rods in the plane can be adjusted to make the intersection point of the axis coincide with the gravity center of the test model. In this way, the additional bending moment caused by axial force can be avoided, resulting in interference with the pitch moment channel and axial force channel. The pitching moment of the model is measured separately by the pitching moment elements.

**Figure 5:**
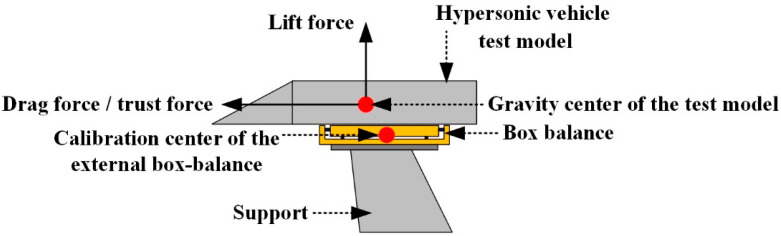
Three-dimensional model diagram of external box-balance.

For the AFMS proposed in this paper, since the axis intersection point of the two-force rods coincides with the gravity center of the aircraft model (as shown in Figure 4), the additional moment introduced by drag and lift is avoided, which is one of the advantages of the AFMS. To verify this point, a mature commercial software called ANSYS was used to carry out virtual static calibration for the AFMS and a mature external box-balance (the calibration center does not coincide with the aircraft test model center of gravity), respectively, as shown in Figure 5. The additional bending moment generated by the AFMS and external box-balance under the action of the same axial force was compared and analyzed. Using the calibration load shown in Table 1, the axial force of 1600 N was applied, respectively, to the gravity center of the aircraft test model in the AFMS force measurement system and to the external box-balance force measurement system. From this, the strain at the sticking position of the strain gauge in the pitching moment force measuring elements was extracted, and, finally, the additional bending moment caused by axial force was calculated (see Table 1). The additional bending moment generated by axial force in the AFMS and external balance was 24.37 N·m and 0.39 N·m, respectively. The analysis results showed that the additional bending moment generated by axial force in the AFMS, which can be almost ignored, was much smaller than that in external box-balance. This was due to the fact that the external box-balance was installed outside the aircraft test model, and the balance calibration center does not coincide with the gravity center of the aircraft test model. This allowed the AFMS to effectively overcome the additional disturbance torque caused by the non-coincidence between the calibration center of the traditional box-balance and the gravity center of the aircraft test model.

**Figure 6:**
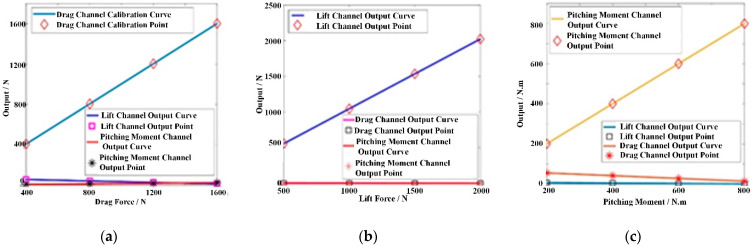
Calibration results for each test channel in the AFMS. (**a**) Drag Force Channel Calibration Curve. (**b**) Lift Force Channel Calibration Curve. (**c**) Pitch Moment Channel Calibration Curve.

In addition, the static calibration of the AFMS was carried out by ANSYS, and the calibration results are shown in Figure 6. The results show that each test channel of the AFMS had excellent linearity, and the interference between the test channels was very small.

## 4. Nonlinear Suppression of Force Measurement System under Vibration State

(a) Analysis of time-varying mechanism of joint stiffness under vibration environment

**Figure 7:**
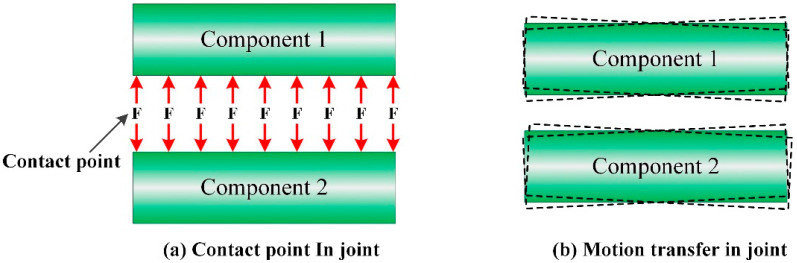
The position of contact point in joint.

When the hypersonic wind tunnel starts, the high-speed transient air flow induces the transient vibration of the force measuring system. Since the effective test time of the hypersonic wind tunnel is only 1~300 ms, it is difficult to completely attenuate vibration in such a short time. The vibration will make the position of the contact point in the joint (the contact surface between the AFMS and the aircraft model) of the force measuring system change randomly, as shown in Figure 7. According to the relevant theories of structural mechanics, the normal force per unit magnitude acts on any point in the connection surface between the AFMS and the test model (denoted as *F_n_*), and the output signal of each test channel in the AFMS is denoted as $V_{Fx},V_{Fy},V_{Fz},V_{Mx},V_{My},V_{Mz}$. The mathematical relationship between them is shown in Formula (1). As the AFMS is a variable cross-section structure, the stiffness between the position of loading point and the sticking position of strain gauge changes with the position of the loading point, meaning the aerodynamic force received by the aircraft model is transmitted through the joint (the connection part between the aircraft model and the AFMS). Therefore, in the process of a hypersonic wind tunnel experiment, the vibration of the force measuring system will lead to changes of the contact point of the joint between the aircraft test model and the AFMS at any time. Based on Formula (1), this will lead to time-varying characteristics of the stiffness of the joint and will make the overall stiffness of the AFMS force measuring system present time-varying characteristics. In this case, if the static calibration force formula is used to identify the aerodynamic force of the aircraft test model, there will be errors.

(1) $$\{\begin{array}{c} △V_{Fx}=e_{11}\cdot F_{1}+e_{12}\cdot F_{2}+\cdots +e_{1n}\cdot F_{n}\\ △V_{Fy}=e_{21}\cdot F_{1}+e_{22}\cdot F_{2}+\cdots +e_{2n}\cdot F_{n}\\ △V_{Fz}=e_{31}\cdot F_{1}+e_{32}\cdot F_{2}+\cdots +e_{3n}\cdot F_{n}\\ △V_{Mx}=e_{41}\cdot F_{1}+e_{42}\cdot F_{2}+\cdots +e_{4n}\cdot F_{n}\\ △V_{My}=e_{51}\cdot F_{1}+e_{52}\cdot F_{2}+\cdots +e_{5n}\cdot F_{n}\\ △V_{Mz}=e_{61}\cdot F_{1}+e_{62}\cdot F_{2}+\cdots +e_{6n}\cdot F_{n} \end{array}$$

**Figure 8:**
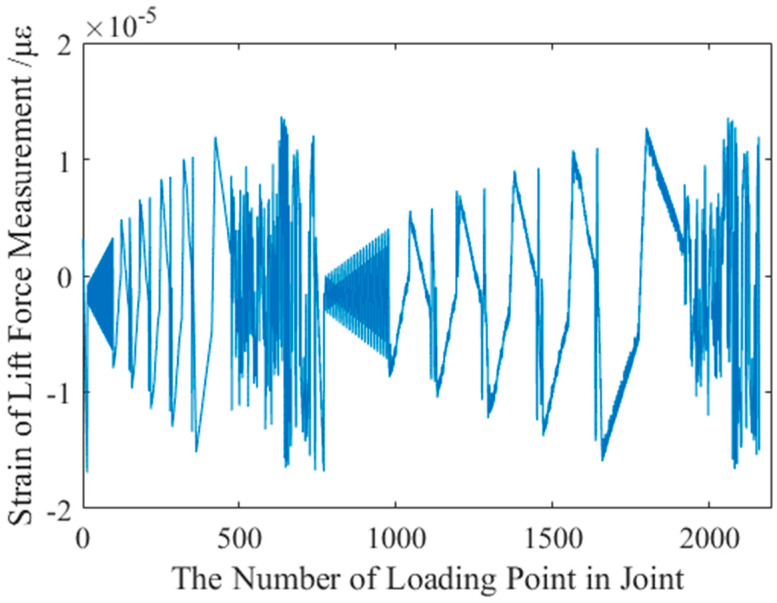
Analysis of the transmission characteristics of the joint.

In order to verify the above point of view, ANSYS was used to analyze the sensitivity coefficients of different positions in the joint of the AFMS, with respect to each force measuring element. The analysis results are shown in Figure 8, and the abscissa in the figure represents the number of loading points at different positions in the joint. The Y-coordinate represents the strain at the sticking position of the strain gauge in the lift force test unit. Through observation, it can be found that when the same force acts on different positions of the joint of the AFMS, the strains at the sticking position of strain gauge in the lift force test unit are different. This result indicates that the stiffness of different positions in the joint of AFMS is different from that of the same lift force test channel.

**Figure 9:**
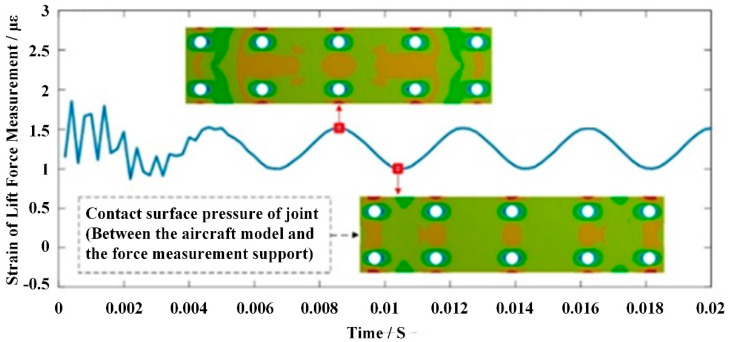
Vibration response of normal force measuring element.

The normal sinusoidal excitation force ($F_{n}=-2460*\sin\left(3000\pi t\right)$) was applied to the gravity center of aircraft test model to simulate the vibration state of the aircraft test model in the pulse wind tunnel test. Figure 9 represents the vibrational response of the lift force measuring elements in the AFMS. This figure shows the contact surface pressure distribution of the AFMS joint at different times. It can be found that the pressure of contact surface of the joint is always changed during the vibration of the aircraft test model. Therefore, the contact point of the joint changes from time to time, resulting in the change of the stiffness of the joint.

To summarize, under vibration conditions, the nonlinearity of the joint causes the stiffness time change of the AFMS. If the static calibration formula is used to identify the aerodynamic force of the model under vibration state, it will inevitably produce errors.

(b) Nonlinear suppression of joint by convex structure

In order to solve the stiffness time varying problem of the joint in the vibration environment on the test accuracy, the convex structures (as shown in Figure 1c) are added at the bolt position of the joints, so that no matter how the joints vibrate, the aerodynamic force acting on the aircraft test model can only be transferred to the force measuring elements through the convex structures. Therefore, the time-varying of the force measurement system caused by the random change of the contact point (force transfer position) in the vibration process are avoided.

In order to verify the above point of view, a mature commercial software, ANSYS, was used to analyze the aerodynamic identification accuracy of the AFMS with and without a convex structure under the same vibration state. The specific approach was as follows: (1) In order to simulate the vibration state of aerodynamic test system in hypersonic wind tunnel, an exciting force of $F_{n}=-2460*\sin\left(3000\pi t\right)$ was applied to the gravity center of the aircraft test model in the AFMS with and without a convex structure, respectively; (2) A lift force of 10,000 N was applied to the gravity center of the aircraft test model in the AFMS with and without a convex structure, respectively; (3) The mature dynamic analysis module in ANSYS was used to analyze the stress distribution; (4) The strains at the sticking position of strain gauge in the lift force test elements of the AFMS with and without convex structure were extracted, respectively, and the lift force received by the AFMS under vibration state was calculated, respectively (see Table 2). 

The lift force identified by the AFMS without and with the convex structure in vibration state was 9670.5 N and 9847.8 N, respectively, and their identification accuracy was 96.71% and 98.48%, respectively. The result shows that the test precision of the AFMS with convex is higher than that of the AFMS without convex. The results also indicate that the convex structure in the joint of the AFMS can effectively suppress the influence of joint stiffness time variation on aerodynamic test accuracy under vibration state.

## 5. Dimensions of BSD

According to the relevant theories of structural mechanics, the mechanical model of the BSD in the wind tunnel test can be equivalent to the mechanical model of the aircraft with a fixed center of gravity and two ends of the model suspended. When the weight and scale of the aircraft test model continue to increase, the support stiffness of the cantilever beam becomes weaker and weaker, meaning the stiffness of the BSD will become weaker and weaker. Therefore, this will lead to an increasing vibration intensity of the aircraft test model and an increasing amplitude of the model during the wind tunnel test. As the transient airflow produces a transient impact on the aircraft test model during the startup process of the hypersonic wind tunnel, inducing transient vibration of the test model, and because the effective test time of the wind tunnel is only 160 ms, the vibration of the test model is difficult to dampen completely in such a short time. When the vibration intensity of the aircraft test model becomes larger and larger, the flow field disturbance caused by the test model vibration will also become larger and larger, which ultimately affects the accuracy of the wind tunnel test. Therefore, for a well-designed BSD, clarifying the scale and weight limits of the aircraft test model can effectively ensure the accuracy of the hypersonic wind tunnel test.

This study proposes a basis that the maximum vibration amplitude of the axis of the aircraft test model around its gravity center be no greater than 1 degree, as this is used to determine the maximum size and weight of the aircraft test model that the BSD can bear. In summary, for the BSD designed in this study, the maximum size and weight of the aircraft model that it can bear are shown in Table 3 and Table 4.

## 6. Conclusions

(1) The additional bending moments, which were generated by the same axial force in the AFMS and a mature external balance (the calibration center does not coincide with the gravity center of aircraft test model), were analyzed and compared. The results showed that the additional bending moment generated by 1600 N axial force in the AFMS and external balance were 24.37 N·m and 0.39 N·m, respectively. The additional bending moment generated in the AFMS were much smaller than that in external box-balance and can be almost ignored. This is because external balance is installed outside the aircraft test model, and the balance calibration center does not coincide with the gravity center of the aircraft test model. The intersection of the two-force rods axes coincided with the gravity center of the test model, which avoided the additional bending moment caused by the axial force and improved the test accuracy. Therefore, the AFMS proposed in this study can effectively overcome the additional bending moment interference;
(2) The analysis results showed that the stiffness of the AFMS joints was different at different positions relative to the same test channel. Similarly, in the vibration environment, the stiffness of the joint changed all the time. Therefore, in the vibration state, the time-varying characteristics of the stiffness of the joint in the AFMS eventually led to the time-varying stiffness of the whole AFMS force measuring system, which showed that the influence of the time-varying stiffness of the joint on the test accuracy cannot be ignored;
(3) The verification results showed that under the same vibration state, the lift force identification accuracy of the AFMS without and with convex structure was 96.71% and 98.48, respectively, which indicated the convex structure can effectively improve the time-varying stiffness of the whole force measuring system under the vibration state, so as to improve the precision of the whole force measuring system.

## Figures and Tables

**Table 1:** Interference analysis among test channels of the AFMS.

	Lift Test Channel/N	Drag Test Channel/N	Pitching Moment Test Channel/N·m
Calibration load	0	1600	0
AFMS	13.56	1601.84	0.39
External box-balance	12.69	1601.86	24.37

**Table 2:** Measurement accuracy of force support under vibration environment.

	AFMS without Convex	AFMS with Convex
Lift force acting on aircraft test model	−10,000 N	−10,000 N
Lift force test channel	−9670.5 N	−9847.8 N
Precision	96.71%	98.48%

**Table 3:** Limit size of aircraft test models allowed by BSD.

The Length of the Aircraft Test Model	The Width of the Aircraft Test Model	The Altitude of the Aircraft Test Model
4800 mm	600 mm	300 mm

**Table 4:** Limit weight of aircraft test model permitted by BSD.

	Limit Weight
Aircraft test model	800 Kg

## Data Availability

No datasets were generated during this investigation.
